# 

**Published:** 1910-08-06

**Authors:** 


### Appointments and Resignations.

Dr. E. Boyd has been appointed medical officer of health under the Stamford Town Council.

Dr. J. E. Barton, for thirty-six years medical officer of the Surrey Council and latterly medical superintendent of the Brookwood Asylum, has resigned.

Dr. Edward C. Seaton's resignation of the position of county medical officer for Surrey, which he has held for nearly twenty years, has been accepted, and he has been appointed consulting medical officer of health; while Dr. T. Henry Jones, the present assistant medical officer for education purposes, has been appointed his successor.

### Jottings.

The King and Queen have made a further gift to the Patients' Library of St. Mary's Hospital.

The King has become patron of the Royal Free Hospital, and the Home Hospitals Association, 16 Fitzroy Square.

Princess Alexander of Teck received last week purses on behalf of the Gravesend Hospital, which contained over £800.

The King has graciously signified his pleasure in becoming Patron of the Royal Hospital for Incurables, Putney Heath, an office held by his late Majesty, King Edward.

A county meeting was convened last week by the Marquis of Hertford at Warwick to consider the question of raising a memorial to King Edward. Lord Hertford announced that Mr. Arthur James, of Rugby, promised at least £500, the Countess of Warwick £100, and Mr. Smith-Ryland £100. A resolution recommending that the memorial should be a hospital for consumptives was carried, and a committee has been formed to carry out the scheme. Birmingham, it is understood, will support the county scheme.

### Gifts and Bequests.

The Rev. Henry Knott Venn, of Honiton, Devon, formerly Rural Dean of Dunkeswell and Honiton, has left £250 to the Devon and Exeter Hospital.

Mr. George Gilyott Morfitt, of Hull, has bequeathed £2,500 to the Hull Royal Infirmary, upon trust to form a fund to be known as the "George Gilyott Morfitt and Fanny Morfitt Fund," to maintain two or more beds, and £500 to the Hull and Sculcoats Dispensary.

Mr. Enoch Hendy, of Bristol, coal merchant, has bequeathed £2,223 each to the Convalescent Home at Durdham Down, Bristol, the Bristol Royal Infirmary, and the Bristol General Hospital, and £556 to the Brompton Hospital for Consumption and Diseases of the Chest. The residue of his property he left equally between the following, among other, institutions in Bristol: The Bristol Eye Dispensary, Orchard Street; the Bristol Eye Hospital, Lower Maudlin Street; the Bristol Royal Hospital for Sick Children and Women, St. Michael's Hill; and the Dispensary, Castle Green.

